# Treatment of Pancreatic Neuroendocrine Tumors: Beyond Traditional Surgery and Targeted Therapy

**DOI:** 10.3390/jcm14103389

**Published:** 2025-05-13

**Authors:** Khyati Bidani, Angela G. Marinovic, Vishali Moond, Prateek Harne, Arkady Broder, Nirav Thosani

**Affiliations:** 1 Department of Internal Medicine, Saint Peter’s University Hospital/Rutgers, New Brunswick, NJ 08901, USA; khyatibidani@gmail.com; 2 Department of Internal Medicine, McGovern Medical School, The University of Texas Health Science Center at Houston, Houston, TX 77030, USA; angela.g.marinovic@uth.tmc.edu; 3 Department of Gastroenterology & Hepatology, West Virginia University, Morgantown, WV 26506, USA; vishali.moond@gmail.com; 4 Department of Advanced Endoscopy, Allegheny General Hospital, Pittsburgh, PA 15212, USA; prateekharne17@gmail.com; 5 Division of Gastroenterology, Saint Peter’s University Hospital/Robert Wood Johnson Medical School, New Brunswick, NJ 08901, USA; 6 Center for Interventional Gastroenterology at UTHealth (iGUT), Department of Surgery, Division of Endoluminal Surgery and Interventional Gastroenterology, McGovern Medical School at UTHealth, Houston, TX 77030, USA

**Keywords:** pancreatic neuroendocrine tumors (PNETs), somatostatin analogs, peptide receptor radionuclide therapy (PRRT), minimally invasive techniques, targeted therapy, EUS-guided ablation, neoadjuvant and adjuvant therapy, conversion surgery, radiofrequency ablation, microwave ablation, molecular, immunotherapy advancements

## Abstract

Pancreatic neuroendocrine tumors (PNETs) are a rare subset of pancreatic neoplasms with diverse biological behavior and clinical presentations. Traditional treatment approaches, such as surgery and targeted therapies, have significantly improved outcomes. However, advancements in molecular biology, immunotherapy, and minimally invasive techniques have ushered in a new era of treatment possibilities. This manuscript explores the emerging modalities in PNET management, emphasizing the need for a multidisciplinary approach tailored to individual patient profiles.

## 1. Introduction

Pancreatic neuroendocrine tumors (PNETs) are rare malignancies arising from pancreatic islet cells, comprising roughly 1–2% of all pancreatic cancers [1]. PNETs display considerable clinical variability and can range from slow-growing, well-differentiated tumors with a favorable prognosis to highly aggressive forms with poor outcomes. PNETs are generally classified into two groups: functional tumors, which produce hormones and lead to distinct clinical syndromes, and nonfunctional tumors, which lack hormonal secretion and are often diagnosed incidentally or due to nonspecific symptoms. Their incidence has been rising, largely attributed to the increased use of cross-sectional imaging modalities, which allow for earlier and incidental detection [2].

Surgical resection remains the gold standard for localized PNETs, offering the only potential cure. However, not all patients are suitable surgical candidates due to the tumor’s location, the extent of disease, or underlying comorbidities [3,4]. Additionally, pancreatic surgeries such as the Whipple procedure, distal pancreatectomy, and total pancreatectomy are associated with significant morbidity, including pancreatic fistula formation, delayed gastric emptying, endocrine and exocrine insufficiency, and prolonged recovery times [5]. Given these risks, alternative treatment strategies have gained prominence, particularly for patients with metastatic or inoperable tumors.

The management of PNETs is increasingly multidisciplinary, integrating systemic therapies and emerging locoregional techniques tailored to the tumor biology, disease stage, and patient-specific factors. Beyond traditional chemotherapy and targeted therapies, approaches such as peptide receptor radionuclide therapy (PRRT), trans-arterial embolization (TAE), trans-arterial chemoembolization (TACE), and radioembolization (TARE) have demonstrated improved tumor control while minimizing systemic toxicity [6]. Additionally, minimally invasive techniques such as radiofrequency ablation (RFA), microwave ablation (MWA), and ethanol ablation (EA) have emerged as promising alternatives for patients who are not candidates for surgery [6].

Despite these advancements, challenges persist in optimizing patient selection, treatment sequencing, and integrating novel therapies. The emergence of immunotherapy and molecular-targeted agents presents new therapeutic possibilities, but their precise role in PNET management remains under investigation. Given the growing complexity of treatment paradigms, an evidence-based, multimodal strategy is essential to maximize efficacy while maintaining safety and patients’ quality of life.

This systematic review examines the current and emerging treatment options for PNETs, providing a comprehensive overview of surgical, systemic, and interventional therapies. It aims to highlight recent advancements, discuss ongoing challenges, and emphasize the importance of individualized patient management in this evolving field.

- **Brief overview of PNETs**

Pancreatic neuroendocrine tumors (PNETs) are broadly classified into functional and non-functional types based on their hormonal activity. Functional PNETs (F-PNETs) secrete bioactive hormones, often resulting in distinct clinical syndromes. In contrast, non-functional PNETs (NF-PNETs) either secrete hormones without clinical symptoms or do not produce hormones at all. The majority of diagnosed pancreatic neuroendocrine tumors (PNETs) are non-functional, with the reported prevalence reaching up to 90% [7]. These tumors are often detected incidentally or present with nonspecific symptoms related to mass effect or metastatic spread. While most PNETs are sporadic, a subset arises in the context of inherited syndromes. The most commonly associated hereditary conditions include multiple endocrine neoplasia type 1 (MEN1) and von Hippel–Lindau (VHL) syndrome, with rarer associations seen in neurofibromatosis type 1 (NF1) and tuberous sclerosis complex (TSC1 and TSC2) [8].

According to the World Health Organization (WHO) 2017 classification, neuroendocrine neoplasms (NENs) are categorized based on their degree of differentiation, mitotic activity, and Ki-67 proliferation index, which together inform both grading and prognosis (Table 1).

Lymph node (LN) metastasis is a significant prognostic factor in PNETs. The likelihood of LN involvement increases with tumor size (>2 cm), higher grade (G2–G3), and functional status [9,10]. F-PNETs such as gastrinomas and VIPomas, even when small (<2 cm), have a higher predilection for LN metastasis and warrant more aggressive surgical exploration [11]. In contrast, small NF-PNETs (<1–2 cm, G1) often exhibit indolent behavior, and a non-operative approach may be considered in select cases after thorough risk assessment [11].

The management of pancreatic neuroendocrine tumors (PNETs) is guided by an integrated approach involving the tumor size, grade, functional status, and extent of metastatic spread. A comprehensive multidisciplinary evaluation is essential and includes biochemical and endocrine work-up to confirm functional status, cross-sectional and functional imaging (CT, MRI, or Ga-68 DOTATATE PET) to assess the disease extent and exclude metastasis, and endoscopic ultrasound (EUS)-guided core biopsy to establish the histological grade [12,13,14]. Following this diagnostic work-up, the current standard of care for PNETs is outlined in Table 2 and Table 3.

Emerging data from the ASPEN trial (Assessment of Surveillance versus Pancreatectomy for Neuroendocrine Tumors) have provided critical insights into the safety of non-operative management for small non-functional pancreatic neuroendocrine tumors (NF-PNETs). This prospective, international, multicenter observational study evaluated patients with NF-PNETs ≤ 2 cm without signs of aggressive behavior on imaging or biopsy. Preliminary findings suggest that active surveillance is a safe and effective strategy in appropriately selected patients, with low rates of progression, metastasis, or delayed complications during follow-up. The ASPEN trial supports the notion that routine surgical resection may not be necessary for all small NF-PNETs, especially those that are low-grade (G1), asymptomatic, and stable on imaging. These findings have influenced current guidelines and clinical practice by reinforcing risk-adapted, individualized decision-making and by expanding the role of conservative management in select patients with indolent tumors [15].

This review will further explore the established treatment strategies, as well as emerging therapies.

## 2. Current Standard Treatments

### 2.1. Surgery

Surgical resection remains the cornerstone for localized PNETs, offering the only curative option. Techniques include enucleation, distal pancreatectomy, and the Whipple procedure. While effective, surgery is limited to early-stage disease and is often contraindicated in metastatic settings.

#### 2.1.1. Potentially Curative Surgery

The choice of surgical procedure for pancreatic neuroendocrine tumors (pNETs) depends on several factors, including the tumor’s location, size, and functionality (functioning vs. non-functioning). Surgical options include enucleation, central pancreatectomy, distal pancreatectomy, Whipple’s procedure, and total pancreatectomy (Table 4). Minimally invasive approaches, such as laparoscopic and robotic techniques, have gained popularity due to their association with improved postoperative outcomes and survival [16].

- **Enucleation**

Enucleation is a parenchyma-sparing approach best suited for small, well-demarcated PNETs that are superficial and safely distant from the main pancreatic duct. Ideal candidates include insulinomas and other functional PNETs ≤ 2 cm (which require resection for symptom control) and select non-functional PNETs that are low-grade (G1) and <2 cm [14]. Enucleation can also be considered in patients with MEN1 who have multiple small tumors, to remove symptomatic or growing lesions while preserving as much pancreatic parenchyma as possible [12]. However, enucleation is typically avoided for tumors suspected to have lymph node metastases or invasive features (e.g., size > 2–3 cm, high grade, or abutting the main duct), in which case a formal resection with lymphadenectomy is preferred [14].

The procedure can be performed via open, laparoscopic, or robotic-assisted approaches. Intraoperative ultrasound is used to determine proximity to the main pancreatic duct (MPD), with a preferred tumor-to-duct distance of ≥2–3 mm [17]. Robotic-assisted enucleation has been shown to enhance precision, particularly in deep or anatomically challenging locations, and is associated with reduced conversion rates and improved visualization. Importantly, recent advances in minimally invasive enucleation (laparoscopic or robotic) have expanded the indications to include tumors near the MPD. Li et al. (2023) reported that even tumors involving or abutting the MPD can be safely enucleated using robotic-assisted techniques and intraoperative MPD support tubes [18].

When appropriately applied, enucleation achieves excellent oncologic outcomes for small, low-grade PNETs. The complete enucleation of benign insulinomas is typically curative, and long-term survival for low-grade tumors is high. Studies show that recurrence rates after enucleation are low and comparable to formal resection in carefully selected patients [19]. However, the absence of lymphadenectomy may miss occult nodal metastases, which occur in up to 10–20% of small NF-PNETs, necessitating careful preoperative staging. Patients’ quality of life post-enucleation is generally favorable, with most patients maintaining pancreatic endocrine/exocrine function [20].

The primary complication is postoperative pancreatic fistula (POPF), with a higher incidence than formal resection (RR~1.5) but typically low-grade and manageable [19]. The rates of new-onset diabetes and exocrine insufficiency are significantly lower than after distal pancreatectomy or Whipple. Enucleation is associated with lower morbidity and the superior preservation of function compared to formal resections, with similar oncologic outcomes for appropriate small tumors [17,19]. International guidelines support its use in selected cases, while Japanese guidelines have traditionally favored resection; recent trends support more conservative approaches in small G1 PNETs [13,21].

Emerging techniques—robotics, ICG fluorescence imaging, and novel sealants—aim to reduce POPF rates and expand indications. Molecular tools like the NETest may help stratify tumors for enucleation vs. resection [22,23]. In MEN1, ongoing studies are refining the timing and extent of surgery to balance cancer risk and organ preservation [24].

- **Central pancreatectomy**

Central pancreatectomy (CP) is a parenchyma-preserving surgical technique typically used for small, benign, or low-grade tumors located in the pancreatic neck or proximal body, particularly when enucleation is not feasible and formal resection (e.g., distal pancreatectomy or pancreaticoduodenectomy) would remove significant healthy parenchyma [25]. It is most commonly considered for non-functional PNETs ≤ 2–3 cm, G1, without radiologic signs of lymph node metastasis or ductal invasion [8].

Central pancreatectomy involves resection of the mid-portion of the pancreas while preserving both the head and tail. The procedure requires two anastomoses: a pancreaticojejunostomy or pancreaticogastrostomy for the proximal remnant, and often closure or drainage of the distal stump. Unlike distal pancreatectomy, CP preserves the spleen and distal pancreas, retaining more endocrine and exocrine function. While CP can be performed through open, laparoscopic, or robotic techniques, it remains technically demanding due to the need for two anastomoses and proximity to the portal and superior mesenteric veins. Intraoperative ultrasound and careful dissection are essential to avoid injury to vascular structures [14].

The major advantage of CP is functional preservation. Studies report significantly lower rates of new-onset diabetes (as low as 5–10%) and a reduced incidence of exocrine insufficiency compared to distal pancreatectomy, especially in younger patients with a long life expectancy [26]. However, CP carries a higher risk of postoperative pancreatic fistula (POPF) compared to other pancreatic resections, due to the creation of a pancreatic-enteric anastomosis in a soft, non-dilated duct and the presence of two pancreatic remnants. POPF rates range between 20 and 60%, though most are low grade and manageable [27]. The long-term oncologic outcomes for appropriately selected PNETs are favorable, provided the tumors are well-differentiated and node-negative [28]. However, CP does not include lymphadenectomy, which limits its utility for higher-grade or suspicious tumors where nodal assessment is essential [14].

Compared to distal pancreatectomy, CP offers the better preservation of pancreatic endocrine and exocrine function but is associated with a significantly longer operative time, prolonged hospital stay, and higher overall and severe morbidity—including a greater risk of clinically relevant pancreatic fistula due to the need for anastomosis in soft pancreatic tissue [26]. In contrast to enucleation, CP allows for the clear margin resection of tumors not amenable to enucleation due to their depth or ductal proximity, though it lacks the oncologic completeness of formal resection with lymphadenectomy [12].

- **Distal pancreatectomy (+/− splenectomy)**

Distal pancreatectomy (DP) is the standard surgical approach for pancreatic neuroendocrine tumors (PNETs) located in the pancreatic body and tail. It is typically indicated for non-functional PNETs > 2 cm or smaller lesions that are intermediate-grade or exhibit growth, where formal oncologic resection with lymphadenectomy is warranted [13,14]. All functional PNETs in the tail (e.g., insulinomas, glucagonomas, VIPomas) generally require resection regardless of size to achieve symptom control and potential cure [12]. In MEN1 patients, distal pancreatectomy may be combined with the enucleation of smaller head lesions to address dominant or symptomatic tumors in the body–tail while preserving pancreatic function [14,20]. Spleen-preserving DP is an option for benign or very low-grade tumors, while splenectomy is preferred when lymphadenectomy of the splenic hilum and artery is required for oncologic clearance [12].

Distal pancreatectomy can be performed via open or minimally invasive (laparoscopic or robotic) approaches. Minimally invasive techniques are increasingly preferred due to shorter hospital stays, lower blood loss, and comparable oncologic outcomes [12,20]. Spleen-preserving distal pancreatectomy may be feasible for low-risk tumors and reduces the risk of post-splenectomy infections [20]. However, oncologic DP typically involves en bloc splenectomy for adequate nodal dissection along the splenic artery and hilum [13].

The complete R0 resection of G1/G2 tumors via DP achieves excellent oncologic outcomes. The five-year disease-specific survival exceeds 80–90% for well-differentiated tumors without metastasis [29]. For tumors > 2 cm, the lymph node metastasis rates approach 30%, reinforcing the need for nodal clearance [30]. Patients with nodal involvement have worse recurrence-free and overall survival compared to node-negative cases [13,14]. Long-term pancreatic function is generally well-preserved. The pancreatic head, which contains the majority of islet cells, remains intact after DP, resulting in a lower incidence of new-onset diabetes compared to pancreaticoduodenectomy [12,19].

The most common complication following distal pancreatectomy is postoperative pancreatic fistula (POPF), occurring in up to 30% of cases, though most are low-grade and can be managed conservatively with drainage and supportive care. The incidence of new-onset diabetes mellitus (NODM) after DP ranges between 5 and 42%, depending on the volume of pancreas removed and patient factors such as pre-existing insulin resistance [31]. However, the risk of NODM is generally lower than after pancreaticoduodenectomy (PD), since the pancreatic head (which contains a larger islet cell population) is preserved. Exocrine insufficiency can occur but is less common, especially in spleen-preserving or functionally parenchyma-sparing variants of DP. A study by Hallack et al. reported that 12.6% of patients developed exocrine pancreatic insufficiency requiring pancreatic enzyme replacement therapy following distal pancreatectomy. A study by Hallack et al. reported that 12.6% of patients developed exocrine pancreatic insufficiency requiring pancreatic enzyme replacement therapy following distal pancreatectomy [32]. Delayed gastric emptying, intra-abdominal abscesses, and wound infections are other potential but less frequent complications. Minimally invasive approaches (laparoscopic or robotic) have demonstrated reduced surgical morbidity, shorter recovery times, and similar oncologic outcomes compared to open surgery [12].

- **Pancreaticoduodenectomy**

Pancreaticoduodenectomy (PD), or the Whipple procedure, is the standard surgical approach for PNETs located in the head of the pancreas, particularly when tumors are >2 cm, intermediate- to high-grade (G2–G3), or closely associated with the main pancreatic duct or ampulla [12]. It is also indicated for functional PNETs in the head (e.g., gastrinomas or somatostatinomas), which often require resection for symptom control and definitive management [33]. In MEN1 patients, PD may be required for large or symptomatic head lesions, while parenchyma-sparing strategies (e.g., enucleation or central pancreatectomy) may be considered for smaller lesions to preserve function [14,20,34]. In select cases, pancreaticoduodenectomy (PD) may be performed for locally advanced PNETs with vascular involvement, where resection with vascular reconstruction can provide long-term disease control in appropriately selected patients [35].

PD involves resection of the pancreatic head, duodenum, distal bile duct, gallbladder, and often part of the stomach, followed by reconstruction with pancreaticojejunostomy, hepaticojejunostomy, and gastrojejunostomy [36]. It can be performed via open, laparoscopic, or robotic approaches, though minimally invasive PD remains technically demanding and limited to experienced high-volume centers. Preoperative pancreatic protocol CT or MRI and intraoperative ultrasound help determine resectability, guide dissection, and assess vascular involvement [14,28].

When complete (R0) resection is achieved, PD provides excellent outcomes in appropriately selected patients. The five-year overall survival rates exceed 80% for G1–G2 pancreatic neuroendocrine tumors (PNETs) without metastasis, with tumor grade, stage, and LN involvement identified as key prognostic factors [37,38]. Studies have shown that pancreatic neuroendocrine tumors (PNETs) larger than 2 cm exhibit a 33.3% rate of lymph node metastasis, and those located in the head of the pancreas are 2.8 times more likely to have lymph node involvement compared to tumors in the body or tail, supporting the routine use of lymphadenectomy during PD [10].

Pancreaticoduodenectomy (PD) is associated with higher morbidity compared to other pancreatic surgical procedures. Postoperative pancreatic fistula (POPF) is one of the most common complications following pancreaticoduodenectomy (PD), with reported incidence rates varying widely depending on patient and surgical factors. While one study reported a clinically relevant POPF rate of 13% among PD patients, others have found rates as high as 36–49%, particularly in those with a soft pancreatic texture or small pancreatic ducts [39]. Other notable complications include delayed gastric emptying, biliary or pancreatic anastomotic leaks, intra-abdominal abscesses, and postoperative hemorrhage. Additionally, new-onset diabetes and exocrine pancreatic insufficiency are more frequently observed following PD than after distal pancreatectomy or enucleation, largely due to the removal of the islet cell-rich pancreatic head, which plays a critical role in endocrine and exocrine function [19,40]. Despite these risks, PD provides comprehensive oncologic clearance, including formal lymphadenectomy, and remains the procedure of choice for tumors in the pancreatic head that are not amenable to conservative resection [41].

- **Total pancreatectomy**

Total pancreatectomy (TP) is generally reserved for select cases of pancreatic neuroendocrine tumors (PNETs) where more conservative surgical approaches are not feasible. It may be indicated in patients with diffuse or multifocal disease, particularly in the setting of hereditary syndromes such as MEN1, where multiple tumors involve both the head and tail of the pancreas and cannot be adequately treated with segmental resection or enucleation [42]. TP is also considered when achieving a complete (R0) resection of a locally advanced tumor necessitates the removal of the entire gland, especially if both the head and body/tail are involved or if there is vascular encasement that precludes partial pancreatectomy [43,44]. In rare situations, TP may be performed for recurrent disease in the pancreatic remnant after prior surgery, or for tumors invading critical structures that require en bloc removal of the entire gland for adequate oncologic control [45]. Due to the permanent loss of endocrine and exocrine function, TP is not considered as a first-line treatment for most PNETs, and is avoided unless clearly necessary to achieve oncologic control.

The surgery includes en bloc resection of the entire pancreas, often along with the spleen, duodenum, and distal bile duct, followed by reconstruction similar to pancreaticoduodenectomy. TP may be performed via an open or robotic approach, though the complexity of reconstruction and risks of endocrine insufficiency remain significant considerations [46]. In MEN1, some institutions may opt for subtotal or near-total resection rather than true TP, to minimize metabolic consequences while still managing multifocal disease [47].

TP results in the complete loss of pancreatic endocrine and exocrine function, necessitating lifelong insulin therapy and pancreatic enzyme replacement. The development of brittle diabetes (also called type 3c or pancreatogenic diabetes) is a major challenge, associated with wide glycemic fluctuations and an increased risk of hypoglycemia [48].

Although long-term oncologic outcomes are acceptable when TP is performed for appropriate indications, patients’ quality of life may be significantly impacted by endocrine insufficiency, digestive symptoms, and nutritional compromise [49]. The rates of major surgical complications are comparable to those seen in pancreaticoduodenectomy, including POPF at the anastomotic sites, infections, and delayed gastric emptying, though the risk may vary depending on the surgical approach [50].

Compared to pancreaticoduodenectomy or distal pancreatectomy, TP offers complete disease clearance in multifocal or diffuse PNETs, but at the cost of total pancreatic insufficiency [43]. It eliminates the risk of recurrence in the residual pancreas, which is particularly relevant in MEN1 patients with multiple lesions. However, its metabolic burden, especially insulin-dependent diabetes, often limits its use to last-resort scenarios [51].

#### 2.1.2. Conversion Surgery

Conversion surgery refers to the resection of previously unresectable pancreatic neuroendocrine tumors (PNETs) following a favorable response to systemic therapy [52]. This approach is increasingly recognized as a viable strategy to achieve curative (R0) resection in patients initially considered inoperable due to local vascular invasion or limited metastatic disease [52]. Typical candidates include those with borderline or locally advanced tumors involving major vessels or with oligometastatic liver disease, particularly when tumors demonstrate stable disease or a partial response following systemic therapy such as peptide receptor radionuclide therapy (PRRT), chemotherapy, or targeted agents like everolimus and sunitinib [52,53].

Key considerations for conversion surgery include a careful assessment of the treatment response and surgical resectability. The NEOLUPANET phase II trial demonstrated promising outcomes with neoadjuvant ^177Lu-DOTATATE in patients with high-risk non-functional PanNETs, achieving a partial radiologic response in 58% and stable disease in 42%, with R0 resection achieved in 24 out of 29 patients. Importantly, the trial reported acceptable perioperative outcomes, with a 24% rate of severe postoperative complications and no perioperative mortality, supporting the safety of this approach when performed in experienced centers [53]. However, conversion procedures are often surgically demanding due to treatment-induced fibrosis, inflammation, or altered tissue planes, which may complicate vascular dissection and margin clearance [54]. As such, these surgeries should be performed in high-volume institutions with dedicated multidisciplinary teams [52,55].

The timing of surgery is also critical—ideally performed after a maximum treatment response but before the onset of therapeutic resistance. Imaging may overestimate the resectability, so intraoperative assessment remains essential, particularly in borderline cases [55]. A meta-analysis by Li et al. reported an overall estimated resection rate of 68.2% and R0 resection rate of 60.2% following neoadjuvant therapy for PNETs, highlighting the feasibility and oncologic potential of this approach [52]. Additionally, a retrospective study by Gao et al. compared the outcomes between patients undergoing conversion surgery and those undergoing direct resection, showing improved progression-free survival among the conversion group following matched analysis, further suggesting that preoperative therapy may help optimize patient selection and outcomes [56].

Despite these encouraging findings, comparative effectiveness data remain limited. Active trials like NEOLUPANET (NCT04385992) are helping establish the role of neoadjuvant PRRT in this setting, but more robust prospective studies are needed to define the optimal treatment regimens, patient selection criteria, and timing for surgery. Future directions include the incorporation of functional imaging and molecular biomarkers to better predict therapy response, and the standardization of multidisciplinary care protocols to expand the role of conversion surgery as a potentially curative option for select patients with PNETs.

#### 2.1.3. Palliative Surgery

Palliative surgical approaches are performed in patients with advanced, metastatic, or unresectable pNETs to alleviate symptoms and improve quality of life. These procedures focus on symptom relief and disease management rather than curative treatment. One common palliative intervention is stent placement, which can relieve biliary or duodenal obstruction caused by pNETs. These minimally invasive procedures provide rapid symptom relief and lower the risk of infection compared to more invasive surgeries. Biliary stents are used to treat obstructive jaundice caused by pancreatic tumors in the head of the pancreas. The stent is placed via endoscopic retrograde cholangiopancreatography (ERCP) to keep the bile duct open, relieving jaundice and improving liver function. Duodenal stents are used to alleviate gastric outlet obstruction from pNETs near the duodenum, restoring food passage through the previously occluded duodenum and relieving adverse gastrointestinal symptoms such as nausea, vomiting, and malnutrition [57].

#### 2.1.4. Bypass Surgery

Bypass surgery is another approach to restoring bile flow and allowing the passage of food through the duodenum to alleviate symptoms caused by pNETs. Biliary bypass surgery is performed in patients with pNETs that obstruct the common bile duct, leading to symptoms such as jaundice and pruritus. This procedure creates an alternative route for bile to flow from the liver to the intestines, bypassing the blocked bile duct. Similar to biliary stents, biliary bypass surgery helps reduce jaundice and improve liver function. Studies have shown that the overall survival is significantly improved in patients with unresectable pancreatic cancers who undergo gastrojejunostomy compared to those who receive a duodenal stent (110 days vs. 63 days) [58]. Gastrojejunostomy is commonly used to relieve the symptoms of gastric outlet obstruction caused by pNETs, including nausea, vomiting, and malnutrition, thereby improving nutritional intake. Although these bypass procedures can provide symptomatic relief, they carry a risk of re-obstruction and offer a limited survival benefit. Common complications include biliary leaks, new obstructions, and anastomotic failure [59].

#### 2.1.5. Debulking Surgery for Metastatic PNETs

In cases where pancreatic neuroendocrine tumors (PNETs) have metastasized, particularly to the liver, complete surgical resection is often not feasible. In such instances, cytoreductive or “debulking” surgery may be considered. This involves resecting a significant portion of the tumor burden—typically ≥ 70–90%—with the goal of alleviating symptoms and potentially prolonging survival. Western guidelines, including those from the NCCN and ENETS, support debulking surgery in carefully selected patients, particularly those with hormonally functional tumors or limited hepatic metastases [60]. Retrospective studies from North America and Europe have demonstrated improved outcomes in this setting [61]. A retrospective study by Huang et al. (2023) showed that debulking surgery improved the 5-year overall survival from 37.8% to 87.5% in patients with unresectable metastatic PNETs, comparable to radical resection [61].

However, this approach remains controversial due to the lack of prospective randomized trials confirming a definitive survival benefit. Consequently, practices differ globally. In Japan, current consensus guidelines have largely moved away from debulking surgery for metastatic PNETs, favoring systemic therapy such as targeted agents or peptide receptor radionuclide therapy (PRRT) instead [62]. The Japanese approach emphasizes the absence of Level I evidence for cytoreduction and a preference for less invasive management strategies, especially in the context of nonfunctional tumors.

### 2.2. Targeted Drug Therapy for Pancreatic Neuroendocrine Tumor

Targeted drugs specifically attack cancer cells by focusing on the molecular pathways involved in tumor growth, angiogenesis, and cell survival. The advent of somatostatin analogs (SSAs), tyrosine kinase inhibitors (TKIs), and mammalian target of rapamycin (mTOR) inhibitors has expanded treatment options for advanced PNETs. While these therapies provide disease control, resistance and side effects remain significant challenges.

- **Somatostatin analogs (SSA)**

Somatostatin analogs (SSAs), such as octreotide LAR and lanreotide, are a cornerstone in the management of well-differentiated (G1–G2) pNETs. They are used both for functioning tumors to control hormone-mediated symptoms (e.g., insulinomas, gastrinomas) and for non-functioning tumors to delay disease progression. SSAs are especially indicated in patients with somatostatin receptor (SSTR)-positive tumors, as confirmed by imaging modalities such as Ga-68 DOTATATE PET/CT [63]. In MEN1-associated pNETs, which are typically multifocal and low-grade, SSAs are effective for both functioning and non-functioning tumors, particularly due to high SSTR2/SSTR5 expression. In a pooled analysis of 105 MEN1 patients, stable disease was achieved in over 75%, with objective responses (partial or complete) in 12.7%, mainly among functioning tumors. These results suggest that SSAs may be more effective in MEN1-related pNETs compared to sporadic cases, potentially delaying disease progression and deferring surgery in select individuals. SSAs were also well tolerated in this population [64].

SSAs exert their effects by binding primarily to somatostatin receptor subtypes 2 (SSTR2) and 5 (SSTR5) on neuroendocrine tumor cells. This binding suppresses intracellular signaling pathways, such as cyclic adenosine monophosphate (cAMP) and mitogen-activated protein kinase (MAPK), leading to reduced hormone secretion, the inhibition of angiogenesis, and cytostatic effects on tumor proliferation [63,64].

The PROMID trial (2009), a randomized double-blind placebo-controlled study in metastatic midgut NETs, showed that octreotide LAR significantly prolonged the time to tumor progression (14.3 months vs. 6.0 months; HR 0.34, *p* < 0.001). Although focused on midgut NETs, PROMID established the tumor-stabilizing potential of SSAs [65]. The CLARINET trial (2014), which included enteropancreatic NETs (including pNETs), demonstrated that lanreotide 120 mg every 4 weeks significantly improved patients’ progression-free survival compared to the placebo. Median PFS was not reached in the treatment group when compared to 18 months in the placebo group (HR 0.47; *p* < 0.001), confirming its efficacy in non-functioning, well-differentiated, SSTR-positive pNETs with Ki-67 < 10%. In functioning pNETs, SSAs also provide rapid and durable symptomatic relief by suppressing hormone hypersecretion [64].

SSAs are generally well tolerated. Common side effects include gastrointestinal disturbances (e.g., diarrhea, bloating, flatulence), cholelithiasis (20–30% with long-term use due to bile stasis), and metabolic disturbances such as mild hyperglycemia or insulin resistance. Most adverse events are grade 1–2 and rarely necessitate discontinuation [64].

SSAs are mainly a cytostatic, stabilizing disease, rather than causing tumor shrinkage. They are less effective in high-grade tumors (G3) or those with low or absent SSTR expression. Over time, patients may develop resistance or escape, necessitating treatment escalation to agents like everolimus, sunitinib, or PRRT [63,64]. Compared to targeted therapies (e.g., everolimus, sunitinib) or chemotherapy, SSAs have fewer side effects and are preferred for indolent, well-differentiated, SSTR-positive tumors. However, they have lower objective response rates and are typically used as the first-line therapy in slowly progressive disease [63,64]. SSA-based combination strategies—e.g., with PRRT, mTOR inhibitors, or anti-angiogenic agents—are under active investigation. Personalized treatment guided by SSTR imaging and molecular biomarkers like NETest may further optimize SSA therapy.

- **Sunitinib**

Sunitinib is an oral multi-targeted tyrosine kinase inhibitor (TKI) approved for the treatment of well-differentiated, unresectable, or metastatic pancreatic neuroendocrine tumors (pNETs). It is particularly indicated for patients with progressive disease not amenable to curative surgery, especially when tumors demonstrate angiogenic activity via the VEGF and PDGF signaling pathways [66,67]. Sunitinib targets several tyrosine kinases involved in tumor growth and angiogenesis, including VEGFR1-3, PDGFR-α/β, c-KIT, and FLT3. By blocking these receptors, it inhibits tumor vascularization and proliferation, leading to cytostatic and antiproliferative effects in pNETs [66].

In the pivotal Phase III trial (NCT00428597), sunitinib significantly improved progression-free survival (PFS) compared to the placebo (11.4 vs. 5.5 months by investigator assessment; HR 0.42, *p* < 0.001). A blinded independent central review (BICR) confirmed these findings with a median PFS of 12.6 months for sunitinib vs. 5.8 months for placebo (HR 0.32, *p* < 0.0001) [66].

In a combined analysis of the Phase III and IV trials, the objective response rate (ORR) was 16.7%, with a median PFS of 12.9 months and overall survival (OS) reaching 54.1 months in the Phase IV cohort [67].

Sunitinib is generally well tolerated, with common side effects including diarrhea, cytopenias, hypertension, and hand–foot syndrome. Most are grade 1–2 and manageable with supportive care. Rare serious events include cardiac dysfunction and bleeding, typically occurring early in treatment [67].

Tumor shrinkage with sunitinib is modest (ORR ~16–24%), and resistance can develop over time. Its use is generally limited to patients with progressive disease, and it is not typically effective for high-grade (G3) tumors or poorly differentiated carcinomas. Regular monitoring for toxicity is essential, especially in patients with comorbidities [66,67]. Compared to SSAs, sunitinib offers more potent anti-tumor effects but with a higher adverse event burden. In contrast to everolimus, another targeted agent used in pNETs, sunitinib may have a stronger antiangiogenic profile but similar survival outcomes. Sunitinib is typically reserved for second-line treatment after SSA failure or as a first-line option in progressive, receptor-negative, or symptomatic disease. Ongoing trials are exploring combination regimens (e.g., sunitinib with PRRT or immunotherapy) and the utility of biomarkers (e.g., circulating VEGF or NETest) to predict response.

- **Everolimus**

Everolimus is recommended for progressive, well-differentiated (G1–G2), non-functional pancreatic neuroendocrine tumors (NF-pNETs), particularly in unresectable or metastatic cases [13]. This is supported by Level 1a evidence from the RADIANT-3 trial, which showed a significant progression-free survival benefit (HR 0.35; 95% CI: 0.27–0.45). In clinical practice, everolimus is also considered for SSTR-negative tumors (where PRRT is not feasible) and more aggressive G2 tumors not suitable for SSA monotherapy [68,69]. Without direct comparison trials, the choice between everolimus and sunitinib is typically guided by patient comorbidities and clinician experience. Emerging data also support its use in select well-differentiated G3 tumors with Ki-67 < 55% (Level 3b, Grade B recommendation) [13]. Everolimus can also be considered for tuberous sclerosis complex (TSC)-associated pNETs as mutations in TSC1 or TSC2 result in mTORC1 pathway hyperactivation, making everolimus particularly effective. Though evidence is limited to small studies and case reports, a clinical benefit has been observed in this subset [70].

Everolimus is an oral mTORC1 inhibitor that blocks tumor cell growth and proliferation by disrupting the PI3K/AKT/mTOR pathway. It binds to FKBP-12, forming a complex that inhibits mTORC1, leading to reduced protein synthesis, angiogenesis, and cell cycle progression [71].

The efficacy of everolimus was established in the RADIANT-3 trial, a phase III randomized placebo-controlled study involving 410 patients with advanced pNETs. Everolimus significantly improved median progression-free survival (PFS) to 11.0 months versus 4.6 months with placebo (HR 0.35; 95% CI, 0.27–0.45; *p* < 0.001) [72]. While the objective response rates were modest (~5%), the majority of patients achieved disease stabilization (73% vs. 51% with placebo) [72].

Everolimus is generally well tolerated, but common side effects include stomatitis, rash, diarrhea, fatigue, and infections. Notable grade 3–4 events include anemia, hyperglycemia, and noninfectious pneumonitis, which may require dose adjustment or treatment interruption [72,73]. Overall, dose reductions occurred in ~59% of patients, and 13% discontinued treatment due to adverse events in the RADIANT-3 trial [72].

Despite efficacy in disease stabilization, complete tumor responses with everolimus are rare. Resistance to everolimus, both primary and acquired, is common. Mechanisms include the activation of alternative signaling pathways (e.g., ERK), the feedback upregulation of PI3K/AKT, or mutations affecting mTOR complex components [71]. Efforts are ongoing to overcome resistance through combination therapies (e.g., with antiangiogenics or chemotherapy). Compared to SSAs, everolimus offers a greater antiproliferative benefit but comes with increased toxicity. In contrast to sunitinib, which targets angiogenesis, everolimus acts directly on cell proliferation pathways. Both drugs are used in similar clinical scenarios, and sequencing or combination strategies are under investigation. Preliminary data from the SEQTOR trial suggest no significant difference in 1-year PFS between everolimus-first vs. chemotherapy-first sequencing [13].

Ongoing trials are evaluating everolimus in combination with PRRT, antiangiogenics, and immunotherapy to enhance efficacy [74]. Combination strategies are under study. For example, the STARTER trial in Japan is evaluating lanreotide with everolimus in advanced PNETs, reflecting an interest in dual SSA + mTOR inhibition [75]. In addition, newer-generation mTOR inhibitors with dual mTORC1/2 inhibition are under development to overcome current resistance mechanisms.

- **Belzutifan (Welireg)**

Belzutifan, a first-in-class hypoxia-inducible factor 2α (HIF-2α) inhibitor, is approved for adult patients with von Hippel–Lindau (VHL) disease who require treatment for associated pancreatic neuroendocrine tumors (pNETs), renal cell carcinoma (RCC), or CNS hemangioblastomas, and do not require immediate surgery. Approximately 9–17% of VHL patients develop pNETs, and while often indolent, larger tumors have metastatic potential. Belzutifan offers a systemic alternative to surgery, particularly when lesions are multiple or surgery is high risk [76].

Belzutifan selectively inhibits HIF-2α, preventing its dimerization with HIF-1β. This blocks the transcription of genes involved in angiogenesis, cell proliferation, and survival, which are upregulated in VHL due to the defective degradation of HIF-2α [77].

In the LITESPARK-004 phase 2 study, belzutifan showed robust antitumor activity in VHL-associated pNETs. Among 22 patients with measurable pNETs, the objective response rate (ORR) was 91%, including seven complete responses. The median duration of response (DoR) and progression-free survival (PFS) were not reached at a median follow-up of 37.8 months, indicating a durable benefit. Belzutifan also significantly reduced the linear growth rate of tumors (median: –4.2 mm/year) [76].

Belzutifan was generally well tolerated. The most common treatment-related adverse events (AEs) were anemia, fatigue, dizziness, and nausea. Grade ≥ 3 AEs were infrequent (18%), and no grade 4/5 treatment-related AEs occurred. Long-term use requires monitoring for anemia and reproductive toxicity, and the drug carries a boxed warning for embryo–fetal harm [76].

Belzutifan provides a non-surgical, systemic treatment option for VHL-associated pNETs with a favorable safety profile and high response rate. It may delay or obviate the need for pancreatic surgery, reducing the risk of surgical complications and organ dysfunction. Long-term follow-up studies are ongoing to further define its safety and efficacy. Trials in sporadic pNETs are yet to be conducted, and its role outside VHL remains investigational [76].

### 2.3. Peptide Receptor Radionuclide Therapy (PRRT)

PRRT, using radiolabeled somatostatin analogs such as Lutetium-177-DOTATATE, has shown promise in treating somatostatin receptor-positive PNETs. These radiolabeled somatostatin analogs selectively target tumor cells expressing somatostatin receptors, such as somatostatin receptor subtype 2 (SSTR2). Upon binding to these receptors, the complex undergoes endocytosis into the tumor cell, and Lutetium-177 delivers radiation that induces DNA breaks, leading to tumor cell apoptosis [78]. Clinical trials, including the NETTER-1 Trial, have demonstrated up to a 79% reduction in the risk of disease progression compared to the control group, as well as improved progression-free survival, overall survival, and quality of life, positioning PRRT as a viable treatment option for patients with advanced disease [79]. PRRT has been further explored under the section of Radiotherapy.

## 3. Emerging Therapies

### 3.1. Newer Targeted Therapies

Tyrosine kinase inhibitors (TKIs) targeting angiogenesis-related pathways have become valuable treatment options for patients with progressive pancreatic neuroendocrine tumors (panNETs), particularly after the failure of prior therapies.

Cabozantinib is a multikinase inhibitor targeting VEGFR2, c-MET, RET, and AXL. In the phase 3 CABINET trial (NCT03375320), cabozantinib significantly improved progression-free survival (PFS) in patients with well-differentiated, progressive NETs. The median PFS was tripled in pancreatic NETs and doubled in extrapancreatic NETs compared to the placebo, establishing cabozantinib as a validated treatment option in this setting [80]. Based on these results, the U.S. Food and Drug Administration (FDA) approved cabozantinib on 26 March 2025 for adult and pediatric patients aged 12 years and older with previously treated, unresectable, locally advanced or metastatic, well-differentiated pancreatic and extra-pancreatic neuroendocrine tumors. The safety profile was consistent with prior TKI experience, with manageable adverse events. The ongoing LOLA trial (NCT04427787) is exploring cabozantinib in combination with lanreotide in G2–G3 GEP-NETs and thoracic NETs, with embedded biomarker analysis (e.g., MET, AXL, VEGFR2) performed to optimize patient selection. Cabozantinib now represents a validated treatment option for advanced progressive NETs, particularly after the failure of prior therapies [80,81].

Lenvatinib, which targets VEGFR1–3, FGFR1–4, and other proangiogenic kinases, was evaluated in the phase II TALENT trial (GETNE1509) for patients with advanced grade 1–2 panNETs or gastrointestinal NETs previously treated with targeted therapies. Among 55 patients with panNETs, the objective response rate (ORR) was 44.2%—the highest reported for a targeted agent in this setting. The median PFS was 15.6 months, and the median duration of response was 19.9 months. Most patients required dose reductions due to adverse events, with fatigue, hypertension, and diarrhea being most common [82]. These results highlight lenvatinib as a promising cytoreductive option, particularly for reversing resistance to prior antiangiogenic therapy.

### 3.2. Immunotherapy

Checkpoint inhibitors targeting PD-1, PD-L1, and CTLA-4 are being actively investigated in the treatment of PNETs. Preliminary studies suggest that combining immunotherapy with other modalities may help overcome the immunologically cold microenvironment of PNETs, thereby enhancing treatment efficacy. PD-1 (Programmed Cell Death Protein 1) is a receptor on T cells that dampens the immune response when activated. PD-L1 (Programmed Death-Ligand 1) binds to PD-1 and is often expressed on tumor cells to evade immune detection. CTLA-4 (Cytotoxic T-Lymphocyte Antigen 4) is another inhibitory receptor on T cells that regulates immune activation and response. Checkpoint inhibitors block these inhibitory pathways to reactivate T cells, enhancing cancer cell destruction. Combination therapies, such as combining PD-1 inhibitors (e.g., nivolumab) with CTLA-4 inhibitors (ipilimumab), have shown greater efficacy than monotherapy in patients with PNETs [83].

### 3.3. Novel Molecular Targets

Recent advancements in genomic and transcriptomic profiling have identified actionable mutations in PNETs, including ATRX/DAXX and MEN1. Drugs targeting these pathways, along with epigenetic modulators, represent a growing area of interest. The ATRX (alpha-thalassemia/mental retardation syndrome X-linked) and DAXX (death-domain associated protein) genes are involved in the ATRX/DAXX pathway, which is frequently mutated in pNETs, particularly those that are well differentiated and advanced or metastatic. These genes are key players in chromatin remodeling, and mutations in them can lead to genome instability and faulty transcription. Additionally, these mutations are associated with telomere dysregulation.

Mutations in the ATRX/DAXX pathway are found in approximately 40% of pNETs and are linked to more aggressive forms of the disease. The loss of ATRX and DAXX is particularly characteristic of pancreatic chromosomal instability (CIN) and correlates with shorter patient survival times [84]. Given that these mutations are associated with more aggressive pNETs, targeting this pathway is essential for effective cancer treatment.

The MEN1 (Multiple Endocrine Neoplasia I) gene, another common mutation in pNETs, encodes a protein that regulates DNA repair, transcription, and cell-cycle progression. Loss of this gene, whether sporadic or genetic, leads to unchecked cellular proliferation and cell-cycle progression, as seen in aggressive pNETs. Targeting pathways involving MEN1, such as the mTOR pathway, can be an effective strategy for treating pNETs with this mutation. The use of mTOR inhibitors, such as Everolimus, has shown efficacy in treating pNETs with MEN1 mutations [85].

In addition to genetic mutations, epigenetic changes such as DNA methylation, histone modifications, and alterations in non-coding RNA have been linked to the development of pNETs. These epigenetic alterations can either silence tumor suppressor genes or activate oncogenes. Studies suggest that epigenetic modulators, including DNA methyltransferase inhibitors and histone deacetylase inhibitors, may help reduce the progression of epigenetically driven pNETs in patients with MEN1 [85].

## 4. Other Drugs for Pancreatic Neuroendocrine Tumors

Several medicines can help control symptoms and tumor growth in people with advanced pancreatic neuroendocrine tumors (NETs). These drugs are used mainly when the tumor cannot be removed with surgery.

### 4.1. Chemotherapy

Chemotherapy drugs can be used alone or in combination to treat pancreatic neuroendocrine tumors (pNETs). Commonly used drugs include Temozolomide (Temodar), Capecitabine (Xeloda), Oxaliplatin (Eloxatin), Fluorouracil (5-FU), Cisplatin or Carboplatin, Etoposide (VePesid), and Irinotecan (Camptosar).

Streptozotocin (STZ), typically combined with 5-fluorouracil (5-FU), is a chemotherapy option for progressive, well-differentiated (G1–G2) pNETs, especially in patients with a high tumor burden or symptomatic disease requiring cytoreduction [86]. It works as an alkylating agent, inducing DNA damage and apoptosis, and preferentially accumulates in pancreatic islet cells via GLUT2 transporters [87]. STZ-based regimens achieve response rates of 25–40%, with durable disease control in both functioning and non-functioning tumors [86]. The SEQTOR trial is a phase III study comparing two treatment sequences—everolimus followed by STZ/5-FU versus STZ/5-FU followed by everolimus—in advanced G1–G2 pNETs. Preliminary data showed no significant difference in progression-free survival between sequences, suggesting that both strategies are viable. Translational analyses also revealed distinct molecular subtypes, potentially informing future treatment personalization [88]. STZ is generally well tolerated, though nausea, renal toxicity, and cytopenias require monitoring. It remains a preferred option in Europe when tumor shrinkage is a priority, while targeted agents dominate in other regions. No head-to-head trials exist, so the treatment choice is individualized. Ongoing studies are evaluating its use in combination regimens and predictive biomarkers such as MGMT and Ki-67 [86].

Temozolomide (Temodar) is an oral alkylating chemotherapeutic agent that damages cancer cells by adding an alkyl group, which prevents cell replication and induces cellular apoptosis. It is typically administered in combination with Capecitabine (Xeloda), a prodrug of 5-FU that is converted to its active form primarily in tumor tissues. This conversion enhances the effectiveness of the drug against cancer cells while minimizing systemic toxicity. 5-FU inhibits thymidylate synthase, disrupting DNA synthesis, halting the cell cycle, and promoting apoptosis. It is also incorporated into fluorouridine triphosphate, which is incorporated into RNA, disrupting protein synthesis. Studies have shown that the combination of Temozolomide and Capecitabine (CAPTEM) results in a higher response rate and increased progression-free survival (58.7 months) compared to Temozolomide alone (53.8 months) [89,90]. This combination is thought to be effective due to Capecitabine’s downregulation of methylguanine methyltransferase (MGMT), making tumor cells more susceptible to Temozolomide [91]. This combination is currently the first-line chemotherapy treatment for well-differentiated pNETs.

Oxaliplatin (Eloxatin), a platinum-based chemotherapeutic agent, forms platinum-DNA adducts, causing interstrand and intrastrand DNA crosslinks. This disrupts DNA polymerase activity and leads to cancer cell apoptosis. While Oxaliplatin is traditionally used for colorectal cancer, it has shown effectiveness in treating pNETs, especially in patients who have failed prior treatments like somatostatin analogs [92]. CAPOX (Capecitabine + Oxaliplatin) is often used for non-resectable pNETs that have failed other first-line treatments. This combination acts synergistically, with Oxaliplatin damaging cancer cell DNA and Capecitabine disrupting DNA replication. Although there is limited research specifically on the use of CAPOX for pNETs, studies have demonstrated its effectiveness for well-differentiated NETs following somatostatin analogue therapy and for metastatic NETs [92,93]. Additionally, studies have shown promising results in combining CAPOX with Bevacizumab for metastatic colon cancer [94,95].

FOLFOX (5-FU + Leucovorin + Oxaliplatin) combines 5-FU, which induces apoptosis by inhibiting thymidylate synthase and disrupting DNA synthesis, with Oxaliplatin, a platinum agent that forms platinum–DNA adducts. Leucovorin, a folic acid analogue, enhances the antitumor effects of 5-FU. FOLFOX is particularly effective for aggressive, pre-treated pNETs that have received prior treatment with CAPTEM. However, studies have shown that while FOLFOX achieves meaningful tumor control, the duration of response is relatively short, with a median of only 2 months. This treatment is also associated with significant side effects, including peripheral neuropathy, myelosuppression, and gastrointestinal issues [96].

TEMOX (Temozolomide + Oxaliplatin) synergistically damages tumor cell DNA by methylating and cross-linking the DNA, halting the cell cycle and inducing apoptosis. This combination has not yet been standardized for treating pNETs [97], and further research is needed to establish its efficacy.

Cisplatin and Carboplatin are platinum–DNA adducts leading to intrastrand and interstrand DNA crosslinks, blocking DNA replication forks, halting the cell cycle, and promoting apoptosis. Both agents act very similarly, but Cisplatin has more adverse side effects than Carboplatin, including ototoxicity and nephrotoxicity. These agents are among the most effective treatments for poorly differentiated neuroendocrine carcinomas [98]. Cisplatin and Carboplatin are often combined with Etoposide (VePesid), which is a topoisomerase II inhibitor. Etoposide prevents DNA unwinding and subsequently induces DNA damage and apoptosis. The combination of Cisplatin or Carboplatin with Etoposide is primarily indicated for high-grade or poorly differentiated pNETs [99]. The platinum–agent and etoposide combination has been shown to achieve higher response rates in aggressive neuroendocrine tumors. However, tumor cells can develop resistance to this regimen, currently not making this a first-line treatment [100,101].

Irinotecan (Camptosar), a topoisomerase I inhibitor, induces DNA damage and apoptosis by preventing the re-ligation of single-strand DNA breaks during DNA replication. It is often combined with platinum agents, such as Cisplatin, to treat various solid tumors, including high-grade gastroenteropancreatic neuroendocrine carcinomas [102]. However, this treatment is associated with toxicities such as neutropenia, nausea, vomiting, and diarrhea. In the TOPIC-NEC Phase 3 Randomized Clinical Trial, the median overall survival for patients with digestive neuroendocrine tumors on Etoposide + Cisplatin was 12.5 months, while those on Irinotecan + Cisplatin had a median overall survival of 10.9 months, suggesting that both regimens are first-line treatments for advanced neuroendocrine carcinomas [103].

Further research into the incorporation of other chemotherapeutic agents, such as Temozolomide, into combination therapies is needed to identify optimal treatment strategies for pNETs.

### 4.2. Neoadjuvant Therapy

Neoadjuvant therapy is increasingly being explored in the management of pancreatic neuroendocrine tumors (pNETs), primarily aimed at tumor downstaging to enable potentially curative surgery in patients with initially unresectable, borderline resectable, or oligometastatic disease [104]. Treatment options include systemic chemotherapy, peptide receptor radionuclide therapy (PRRT), and, in some cases, targeted agents. Among chemotherapy regimens, CAPTEM (capecitabine and temozolomide) is most commonly used due to its favorable response rate; in one series, six borderline resectable pNET patients receiving neoadjuvant CAPTEM showed tumor regression (partial response or stable disease), enabling complete surgical resection in all, with negative margins in most [104]. Another study by Ostwal et al. demonstrated partial or stable responses in the majority of 30 patients with locally advanced or liver metastatic disease treated with CAPTEM, further supporting its utility in preoperative settings [104,105]. In contrast, the efficacy of streptozotocin-based regimens appears more modest, with limited radiologic responses observed and an uncertain benefit in tumor downstaging [104].

PRRT using radiolabeled somatostatin analogs (e.g., ^177^Lu-octreotate) has shown potential for shrinking tumors to allow resection. A Dutch study reported that in 29 patients with nonfunctioning pNETs, 31% became resectable following PRRT, with a median progression-free survival (PFS) of 69 months in the resected group compared to 49 months in the unresected cohort [106]. Other retrospective reports, including those by Partelli et al., have noted higher R0 resection rates and lower rates of postoperative complications like pancreatic fistulas when neoadjuvant PRRT preceded surgery [13].

Additionally, chemo-PRRT combinations have been explored, such as sandwich regimens alternating CAPTEM and PRRT cycles. These approaches have shown promising radiologic responses and disease control, though prospective trials are needed [104]. Despite the growing evidence base, the role of neoadjuvant therapy remains investigational. The selection criteria for optimal candidates, timing, and treatment sequencing are areas of ongoing research. Nonetheless, neoadjuvant therapy represents a valuable tool for downstaging disease, improving surgical outcomes, and expanding resectability in select patients with pNETs.

### 4.3. Adjuvant Therapy

The role of adjuvant therapy in the management of resected pancreatic neuroendocrine tumors (PNETs) remains controversial due to conflicting evidence and a lack of high-quality prospective trials. Despite radical resection being the cornerstone of curative treatment for non-metastatic PNETs, recurrence rates remain significant, particularly in intermediate- to high-risk patients.

A large multi-institutional retrospective study by Barrett et al. analyzed 1871 patients with resected GEP-NETs and compared outcomes in 91 stage I–III patients who received adjuvant therapy. The study found that cytotoxic chemotherapy was associated with a significantly lower 5-year recurrence-free survival (36%) compared to 81% in patients who did not receive adjuvant therapy. Importantly, adjuvant therapy conferred no overall survival benefit, and on multivariable analysis, was negatively associated with recurrence-free survival after adjusting for tumor and patient characteristics [107].

These findings are consistent with a separate population-based study by Masi et al., which also showed that adjuvant chemotherapy offered no significant OS benefit across most subgroups. Chemotherapy conferred a survival advantage only in patients with poorly differentiated or high-grade lesions, but not in those with positive nodes, margins, or local invasion. This supports the notion that surgical resection remains the most impactful curative intervention, even in the presence of high-risk pathological features [108].

In contrast, a single-center retrospective study by Gao et al. suggested a potential benefit of adjuvant long-acting octreotide in patients with G2 PNETs. Patients who received octreotide after surgery had significantly higher 2- and 3-year disease-free survival (DFS) rates compared to observation (98.3% vs. 88.7% and 96.6% vs. 85.9%, respectively), with adjusted analyses showing a reduced hazard of recurrence at 3 years (HR 0.2, *p* = 0.044) [109]. However, these results stem from a non-randomized retrospective design and must be interpreted cautiously.

To address this gap, the ongoing SWOG S2104 randomized phase II trial is investigating the role of adjuvant capecitabine and temozolomide (CAPTEM) versus observation in high-risk patients with resected G2–G3 PNETs (Ki-67 up to 55%) and a high Zaidi risk score (≥3). This study represents the first prospective effort to assess cytotoxic chemotherapy in this setting and will be critical in guiding future adjuvant treatment strategies [110].

In summary, routine adjuvant therapy is not currently the standard of care for resected PNETs. Limited retrospective evidence suggests the possible benefit of somatostatin analogues in select G2 tumors, while cytotoxic chemotherapy may only be warranted in poorly differentiated cases. Ongoing prospective trials like S2104 are expected to clarify the utility of adjuvant treatment in high-risk patients.

## 5. Minimally Invasive Techniques

Advances in laparoscopic and robotic surgery enable precise resection with reduced morbidity. Additionally, endoscopic ultrasound-guided techniques, including radiofrequency ablation and ethanol injection, provide non-surgical options for small, localized tumors.

### 5.1. Ablative Treatments (Ablation)

- **Radiofrequency Ablation (RFA):**

Radiofrequency ablation (RFA) has emerged as a promising minimally invasive technique for managing pancreatic neuroendocrine tumors (PNETs) [111,112]. Endoscopic ultrasound-guided RFA (EUS-RFA) offers an effective alternative to surgery, particularly for small, localized tumors, and its precision combined with the ability to spare surrounding tissues has positioned it as an potentially essential tool in the therapeutic repertoire for selected patients [111,112]. EUS-RFA is particularly suited for functional PNETs, such as insulinomas, where the cessation of hormone hypersecretion is the primary goal, and for small (<2 cm) non-functional PNETs when surgery is contraindicated or the patient refuses it [112,113]. A recent meta-analysis by Armellini et al. reports impressive clinical success rates for EUS-RFA, with 95.1% for functional PNETs, defined as significant symptom resolution through the suppression of hormonal hypersecretion, and 93.4% for non-functional PNETs, characterized by complete tumor ablation confirmed on follow-up imaging [114]. Notably, functional PNETs do not require complete ablation to achieve symptomatic relief, as the partial destruction of tumor tissue can suffice to halt hormone production [114].

Two primary devices are utilized for EUS-RFA in PNETs: the STARmed RFA system and the Habib EUS-RFA catheter [112,113]. The STARmed system features a 19G monopolar electrode needle with automatic energy modulation and a unique cooling system. This system incorporates a peristaltic pump that delivers a continuous saline solution to the needle tip, preventing overheating and tissue charring. The saline perfusion creates a controlled thermal environment, allowing for more extensive ablation zones while minimizing the thermal damage caused to surrounding structures such as ducts or vessels [111,112]. This mechanism makes the STARmed system particularly effective for larger or irregularly shaped tumors and ensures safety near critical structures [112,113].

In contrast, the Habib catheter is a smaller, flexible device compatible with both 19G and 22G needles, making it less invasive and suitable for small, well-demarcated lesions or challenging anatomical areas [112,113]. The Habib catheter is also cost-effective, as it utilizes standard electrosurgical generators, reducing costs in resource-limited settings. Additionally, its simpler configuration allows for a quick setup, which is advantageous in centers with existing fine-needle aspiration (FNA) workflows [111,112]. While the STARmed system excels in precision and adaptability, particularly for complex cases, the Habib catheter provides a practical and efficient alternative for straightforward cases. Both systems demonstrate high efficacy, with the choice depending on tumor characteristics, patient needs, and institutional resources.

Despite its high efficacy, EUS-RFA has limitations. The tumor’s size and proximity to critical structures, such as the pancreatic duct, bile duct, or major vessels, can increase the risk of complications and limit the feasibility of the procedure [111,113]. Larger tumors (>2 cm) have shown lower success rates due to incomplete ablation [111,112]. Adverse events, though relatively rare, include mild abdominal pain, transient acute pancreatitis, ductal strictures, and, in rare cases, perforation [112,113]. These risks can be mitigated through the peri-procedural administration of prophylactic antibiotics, rectal indomethacin, and careful patient selection [112,113].

EUS-RFA has a strong potential to complement or even replace surgery in specific cases, especially with ongoing advancements in device technology and imaging [113]. Prospective trials are expected to provide robust comparative data on its safety and long-term outcomes versus surgical resection. Nonetheless, multidisciplinary evaluation remains essential to ensure optimal patient outcomes, particularly for non-functional PNETs, where long-term efficacy and recurrence data are still limited. In conclusion, EUS-RFA is a safe and effective minimally invasive technique for treating small PNETs, with the potential to redefine treatment algorithms for selected patients. However, further studies are needed to refine patient selection criteria and procedural protocols.

- **Microwave Thermotherapy:**

Microwave ablation (MWA) is an emerging minimally invasive technique for treating pancreatic neuroendocrine tumors (PNETs), particularly in patients who are poor surgical candidates or have unresectable lesions [115,116]. Egorov et al. also highlight its potential utility in managing functional PNETs, as it effectively controls symptoms related to hormone hypersecretion [115]. MWA achieves tumor destruction through high-frequency electromagnetic waves, generating rapid heating and uniform coagulative necrosis of the targeted tissue [116,117]. Compared to radiofrequency ablation (RFA), MWA offers advantages such as higher intra-lesion temperatures, shorter ablation times, homogeneous energy deployment, and a reduced susceptibility to the heat sink effect, making it a promising alternative in the management of PNETs [116,117].

Recent studies have demonstrated the safety and efficacy of MWA for PNETs. According to Egorov et al., MWA achieved symptom resolution in all patients with functional PNETs treated for hyperinsulinemia, with an average follow-up of 31 months [115]. While minor complications such as pseudocysts and pancreatic fistulas were observed, no cases of tumor recurrence or persistent hormonal symptoms were reported during follow-up [115]. Similarly, Robles-Medranda et al. reported the successful application of EUS-guided MWA for an unresectable PNET, achieving complete tumor ablation without post-procedural adverse events, with the patient remaining asymptomatic at an 8-month follow-up [117].

The technique offers flexibility in its application, with MWA being performed through laparotomy, percutaneous, or endoscopic ultrasound (EUS)-guided approaches [116]. The EUS-guided approach has been highlighted for its precision and safety, allowing real-time imaging to ensure optimal probe placement and effective energy delivery while minimizing damage to surrounding structures [63]. Furthermore, Ardeshna et al. noted that MWA provides a larger ablation volume and consistent energy transfer, even in difficult-to-access pancreatic lesions [116].

Despite these advantages, the role of MWA in managing PNETs remains under investigation. The lack of large-scale studies and randomized controlled trials limits robust conclusions about its long-term outcomes and comparative efficacy versus RFA or surgery. Current evidence suggests that MWA may be particularly beneficial for patients with small functional PNETs or those with significant surgical risks [115,116,117]. Further research is necessary to refine patient selection criteria, optimize procedural parameters, and evaluate long-term outcomes.

- **Ethanol Ablation:**

Ethanol ablation (EA) is an alternative minimally invasive technique used to manage pancreatic neuroendocrine tumors (PNETs), particularly small, localized lesions in patients who are poor surgical candidates [118]. The procedure involves injecting concentrated ethanol directly into the tumor under endoscopic ultrasound (EUS) guidance, inducing coagulative necrosis to destroy neoplastic tissue [118,119]. EA has shown efficacy in treating functional PNETs, such as insulinomas, by resolving symptoms through hormone suppression, and in achieving the complete ablation of non-functional PNETs, as confirmed by imaging [120]. According to So et al., EA achieved a 65% complete ablation rate and partial response in 21.6% of cases, while Garg et al. reported clinical success rates as high as 82.2% in select patients [119,120].

The EA procedure involves advancing a 22- or 25-gauge EUS needle into the tumor and injecting ethanol incrementally until a hyperechoic blush is visible within the lesion. The injection is carefully monitored to avoid leakage into surrounding tissues, and additional passes are performed for larger tumors or residual areas [64]. Typically, ethanol concentrations range from 95% to 99%, and the total volume injected is adjusted based on the tumor size and location [118,119].

While EA is generally safe, it is associated with risks such as mild acute pancreatitis, abdominal pain, and, in rare cases, necrotizing pancreatitis or fistula formation [118,119]. Garg et al. reported an overall adverse event rate of 11.5%, with pancreatitis being the most common complication, occurring in 7.6% of cases [120]. However, the complication rates for EA are comparable to those of RFA, with no significant differences in safety profiles [120].

EA demonstrates similar clinical success (82.2%) and technical success (96.7%) rates compared to RFA but is considered less technically demanding and more accessible in resource-limited settings [118,120]. The main limitations of EA include variations in technique, a lack of standardized protocols, and limited long-term outcome data. Larger, multicenter trials are needed to establish standardized guidelines for its use and to compare its long-term efficacy with other ablative techniques or surgical resection.

### 5.2. Embolization

Embolization involves blocking the blood supply to the tumor to deprive it of nutrients and oxygen. There are three main types:

- **Arterial Embolization (TAE):**

Trans-arterial embolization (TAE) is a liver-directed therapy for pancreatic neuroendocrine tumors (PNETs), particularly in patients with liver metastases who are not candidates for surgery [121,122]. The procedure involves the selective catheterization of hepatic arteries supplying the tumor, followed by embolic agents that induce ischemia and tumor necrosis [121,123]. TAE is especially effective for hypervascular PNET liver metastases and is endorsed by the National Comprehensive Cancer Network (NCCN) and the European Neuroendocrine Tumor Society (ENETS) [122]. Bai et al. reported a disease control rate (DCR) of 75% and an objective response rate (ORR) of 37.5%, with better progression-free survival (PFS) in pancreatic compared to rectal NENLM [122]. Additionally, Chen et al. found that 65.9% of patients exhibited a tumor response based on the RECIST criteria, while 77.5% responded according to the mRECIST criteria, with responders experiencing significantly improved overall survival (OS) [123].

TAE is performed via percutaneous catheterization, targeting tumor-feeding hepatic arterial branches [121]. Common embolic agents include polyvinyl alcohol (PVA) particles, microspheres, and gelatin sponges, which occlude the arterial supply, leading to ischemia and necrosis [121,122]. Multiple sessions may be required, particularly in cases of high hepatic tumor burden [123].

TAE is generally well tolerated but may cause post-embolization syndrome, presenting as fever, nausea, abdominal pain, and transient liver enzyme elevation [122,123]. Bai et al. reported abdominal pain in 71.9% of patients and transient transaminase elevation in 50%, with rare cases of thrombocytopenia and liver abscess [122]. Chen et al. noted that early TAE (within four months of diagnosis) was associated with better treatment responses and reduced mortality [123].

TAE is often compared to trans-arterial chemoembolization (TACE), which combines embolization with chemotherapy [121]. While TACE may provide additional tumor control, some studies suggest that TAE alone achieves similar outcomes with fewer side effects. Chen et al. found that the tumor response following TAE correlated with an improved OS, highlighting its effectiveness as a standalone therapy [123].

Despite its benefits, TAE is not curative and is best used as part of a multimodal treatment strategy. Further studies are needed to standardize protocols, refine patient selection, and compare its long-term efficacy with other liver-directed therapies like TACE or peptide receptor radionuclide therapy (PRRT) [122]. In conclusion, TAE is a valuable palliative treatment for PNETs with liver metastases, offering effective tumor control and symptom relief with an acceptable safety profile. Ongoing advancements in interventional oncology continue to refine its role in metastatic PNET management.

- **Chemoembolization:**

Trans-arterial chemoembolization (TACE) is a liver-directed therapy used for pancreatic neuroendocrine tumors (PNETs) with liver metastases, particularly in patients ineligible for surgical resection [124]. The procedure combines arterial embolization with localized chemotherapy infusion (e.g., doxorubicin, cisplatin, mitomycin C) to induce tumor ischemia and cytotoxicity [124]. TACE is effective for tumor control and symptom relief, with Touloupas et al. reporting a median overall survival (OS) of 5.3 years and a median time to liver progression (TTLP) of 19.3 months in a 15-year retrospective study of 202 patients [124]. Ngo et al. further demonstrated that TACE conferred a significant survival advantage over radioembolization (TARE), with OS ranging from 16.8 to 81.9 months, compared to 14.5 to 66.8 months for TARE [125].

TACE can be performed as conventional TACE (c-TACE) using lipiodol as a drug carrier or drug-eluting bead TACE (DEB-TACE), where microspheres provide sustained drug release [124]. The procedure is performed selectively (targeting tumor-feeding arteries) or non-selectively (lobar or total hepatic embolization), depending on the tumor burden and distribution [124].

TACE is generally well tolerated but may cause post-embolization syndrome, presenting as fever, nausea, abdominal pain, and transient liver enzyme elevation [124]. Egger et al. reported a 22.6% morbidity rate, with complications such as hepatic abscesses and biliary strictures, while Touloupas et al. found severe complications (Clavien-Dindo grade ≥ 3) in only 3% of cases, with no procedure-related mortality [124,126].

Compared with trans-arterial embolization (TAE) and radioembolization (TARE), TACE may offer better tumor shrinkage, though some studies suggest that TAE achieves similar control with fewer complications [125]. Ngo et al. found that TACE improved OS compared to TARE, but hepatic progression-free survival (PFS) was similar between the two [125].

Despite its benefits, TACE is a palliative treatment rather than curative. Further studies are needed to optimize patient selection and standardize protocols while comparing its long-term efficacy against other liver-directed therapies such as peptide receptor radionuclide therapy (PRRT). In conclusion, TACE remains an essential treatment for PNET patients with liver metastases, providing effective tumor control and symptom relief with an acceptable safety profile. As liver-directed therapies evolve, TACE continues to play a crucial role in multidisciplinary PNET management.

- **Radioembolization:**

Trans-arterial radioembolization (TARE), also known as selective internal radiation therapy (SIRT), is a liver-directed treatment for unresectable pancreatic neuroendocrine tumor (PNET) liver metastases, particularly in patients with progressive disease despite systemic therapy [127]. The procedure involves the intra-arterial infusion of yttrium-90 (^90Y)-labeled microspheres, which selectively lodge in tumor vasculature and emit β-radiation, leading to localized tumor destruction [128]. TARE has demonstrated strong tumor control, with Schaarschmidt et al. reporting an 83.5% disease control rate (DCR) at 3 months and 50.9% at 12 months, and a mean overall survival (OS) of 38.9 months [128]. Wong et al. found a median OS of 33 months, with the OS extending to 42 months in PNET patients compared to 29 months in other neuroendocrine tumors [129].

TARE is a two-step procedure, starting with a technetium-99m macroaggregated albumin (^99mTc-MAA) simulation to assess the hepatic arterial anatomy and lung shunting, followed by ^90Y-microsphere infusion [127]. Microspheres, available as resin (SIR-Spheres^®^, Sirtex Medical, Sydney, Australia) or glass (TheraSphere^®^, Boston Scientific, Marlborough, MA, USA) particles, are selectively delivered via hepatic artery catheterization to optimize the radiation dosing while sparing normal liver tissue [128].

TARE is generally well tolerated, but complications include post-embolization syndrome (fever, nausea, abdominal pain) and hepatic toxicity. Wong et al. reported grade ≥ 3 hepatic toxicity in 7.6% of patients, with new-onset ascites occurring in 5%. Schaarschmidt et al. found that a higher tumor burden and tumor grade correlated with a shorter OS, but extrahepatic metastases did not significantly impact survival [128,129].

Unlike trans-arterial embolization (TAE) and trans-arterial chemoembolization (TACE), which induce ischemia, TARE achieves tumor control through targeted radiation [127]. Wong et al. reported that while TACE provides comparable progression-free survival (PFS), TARE achieves durable tumor control with fewer retreatments [129]. Schaarschmidt et al. found that TARE, as a second-line therapy (after somatostatin analog failure), resulted in longer OS (44.8 months) compared to salvage use (30.6 months) [128].

Although effective, TARE is not curative and is best used as part of a multimodal approach, particularly in combination with peptide receptor radionuclide therapy (PRRT). Further research is needed to refine patient selection, optimize dosimetry, and assess long-term benefits.

### 5.3. Radiation Therapy for Pancreatic Neuroendocrine Tumor

- **External Beam Radiation Therapy:**

External beam radiation therapy (EBRT) is primarily used for symptom control and palliation in unresectable or metastatic pancreatic neuroendocrine tumors (PNETs) [130]. While historically considered radioresistant, modern techniques like intensity-modulated radiation therapy (IMRT) and stereotactic body radiation therapy (SBRT) have shown improved tumor control with minimal toxicity [130]. EBRT is indicated for pain relief, obstruction management, and bone metastases. A systematic review by Chan et al. analyzed 176 patients and reported a 46% radiological response rate in non-surgical patients and a median overall survival (OS) of 9 to 19 months in metastatic cases [130]. The American Cancer Society (ACS) acknowledges EBRT as an effective option for tumor shrinkage and symptom relief [6].

EBRT is delivered using a linear accelerator, with fractionated dosing (50.4 Gy in 28 fractions) or high-dose SBRT [130]. Advanced techniques such as 3D-conformal radiotherapy (3D-CRT), IMRT, and SBRT enable precise targeting while sparing surrounding organs. Chan et al. reported a 57% local tumor response rate with SBRT, suggesting an advantage over conventional fractionation [6].

EBRT is generally well tolerated, but gastrointestinal toxicity remains a concern. Acute side effects include diarrhea, nausea, fatigue, and transient neutropenia, with grade 3+ toxicity occurring in 11% of cases [130]. The ACS notes that careful planning is needed to minimize severe abdominal radiation effects [6].

EBRT provides local tumor control but lacks the systemic benefits of peptide receptor radionuclide therapy (PRRT) [130]. Unlike trans-arterial embolization (TAE) and trans-arterial chemoembolization (TACE), EBRT does not require arterial access, making it suitable for patients with compromised vasculature [130]. However, its impact on survival and disease modification remains uncertain, with Chan et al. highlighting the need for prospective trials. Further research is required to refine dose protocols, patient selection, and combination strategies with systemic therapies like PRRT.

- **Radioembolization:**

Trans-arterial radioembolization (TARE), also known as selective internal radiation therapy (SIRT), is a liver-directed treatment for unresectable PNET liver metastases, particularly in patients with progressive disease despite systemic therapy. It involves the intra-arterial infusion of yttrium-90 (^90Y)-labeled microspheres, which selectively lodge in tumor vasculature and deliver targeted β-radiation to induce tumor destruction. TARE has demonstrated strong tumor control, with studies reporting a median overall survival (OS) of up to 42 months in PNET patients. It is generally well tolerated but may cause post-embolization syndrome and hepatic toxicity. While TARE offers durable tumor control compared to trans-arterial embolization (TAE) and trans-arterial chemoembolization (TACE), it is not curative and is best used in a multimodal approach, including peptide receptor radionuclide therapy (PRRT). For further details on embolization techniques, refer to the embolization section.

- **Peptide Receptor Radionuclide Therapy (PRRT):**

Peptide receptor radionuclide therapy (PRRT) is a targeted treatment for somatostatin receptor-positive (SSTR+) pancreatic neuroendocrine tumors (PNETs), delivering localized radiation via Lutetium-177 dotatate (Lu-177-DOTATATE) to induce tumor necrosis [131]. It is primarily indicated for metastatic, progressive, or unresectable well-differentiated PNETs, particularly in patients who have failed first-line somatostatin analog therapy [132]. While the NETTER-1 trial established PRRT’s efficacy in midgut neuroendocrine tumors, retrospective studies in PNETs have reported a median progression-free survival (PFS) between 20 and 39 months and an overall survival (OS) ranging from 37 to 79 months [133]. The OCLURANDOM trial demonstrated a 12-month PFS rate of 80% for PRRT versus 42% for sunitinib, with a median PFS of 20.7 months compared to 11 months [133]. Additionally, an Italian multicenter study by Pusceddu et al. found that upfront PRRT significantly improved PFS (median 2.5 years) compared to chemotherapy or targeted therapy (0.7 years, HR: 0.35, *p* < 0.001) [131].

PRRT consists of four cycles of Lu-177 DOTATATE (7.4 GBq per cycle) administered every 6–8 weeks. 68Ga-DOTATATE PET/CT imaging is used for patient selection and response prediction [132]. Recent research suggests that dosimetry-guided personalized PRRT may optimize outcomes by tailoring radiation doses to the tumor burden [133].

PRRT is generally well tolerated, with mild nausea, fatigue, and transient bone marrow suppression being the most common side effects. Grade 3–4 hematologic toxicity occurs in 10–20% of patients, and long-term risks include a 1.8% incidence of secondary myelodysplastic syndrome or leukemia [132]. Renal toxicity is mitigated with amino acid infusions during treatment [133].

PRRT offers superior tumor control compared to everolimus and sunitinib, especially in patients with high somatostatin receptor expression. Preliminary results from two major phase III trials—NETTER-2 and COMPETE—support the use of peptide receptor radionuclide therapy (PRRT) in advanced gastroenteropancreatic neuroendocrine tumors (GEP-NETs), including higher-grade and first-line settings. The NETTER-2 trial showed that ^177Lu-DOTATATE significantly prolonged the median progression-free survival (PFS) to 22.8 months compared to 8.5 months with high-dose octreotide LAR alone in newly diagnosed G2/G3 GEP-NETs. Similarly, the COMPETE trial demonstrated that ^177Lu-edotreotide achieved a superior median PFS (23.9 months) compared to everolimus (14.1 months) in patients with progressive, somatostatin receptor-positive GEP-NETs. These findings highlight PRRT’s expanding role beyond traditional later-line treatment [134,135]. Additionally, alpha-emitting PRRT agents (225Ac-DOTATATE) are under study, showing higher tumor penetration and potential efficacy over Lu-177 [133].

PRRT is a highly effective therapy for advanced SSTR-positive PNETs, significantly prolonging PFS while maintaining a favorable safety profile. Future research aims to refine treatment sequencing, personalized dosing, and combination strategies.

## 6. Challenges and Future Directions

The management of pancreatic neuroendocrine tumors (PNETs) has evolved significantly with the introduction of targeted therapies, peptide receptor radionuclide therapy (PRRT), and minimally invasive treatments. Despite these advancements, several challenges persist, including optimal patient selection, resistance to targeted treatments, the limited efficacy of immunotherapy, disparities in access to care, and the need for personalized treatment strategies.

- **Patient Selection for Novel Therapies**

PNETs exhibit substantial heterogeneity in terms of grade (Ki-67 index), differentiation, tumor burden, and functional status, making treatment selection challenging [136,137]. Current guidelines attempt to tailor first-line therapy based on disease factors. For well-differentiated, low-proliferation tumors (Ki-67 < 3%), somatostatin analogs (SSAs) such as octreotide and lanreotide are typically first-line treatments, given their role in slowing tumor progression and controlling symptoms [138]. In contrast, more aggressive tumors with higher Ki-67 indices or rapid growth may warrant the earlier initiation of targeted therapies (e.g., everolimus, sunitinib) or cytotoxic chemotherapy (e.g., capecitabine/temozolomide) [138]. However, refining patient selection further through predictive biomarkers and imaging-based assessments remains an unmet need to optimize treatment outcomes.

- **Resistance to Targeted Therapies**

The approval of targeted agents marked a turning point in advanced PNET treatment. Everolimus (an mTOR inhibitor) and sunitinib (a VEGF receptor tyrosine kinase inhibitor) were shown in phase III trials to significantly prolong PFS in metastatic PNET, establishing these drugs as standard options. However, the objective response rates with these agents are low (typically < 10% tumor shrinkage rates), with most patients achieving disease stabilization rather than tumor regression [137]. More critically, virtually all patients eventually develop disease progression on these therapies, reflecting intrinsic or acquired resistance [80,139,140]. The median PFS for everolimus or sunitinib is in the order of 11–12 months, after which tumor growth resumes in the majority of cases [140]. Acquired resistance to targeted therapy is a major obstacle limiting long-term efficacy [140].

Resistance mechanisms include the compensatory activation of parallel signaling pathways, such as the PI3K-AKT pathway in response to mTOR inhibition, and the hypoxia-driven upregulation of HIF-1α and HIF-2α, which allows tumors to escape VEGF-targeted therapy [86,87]. Additionally, the recruitment of alternative angiogenic factors and pericyte-mediated vascular stabilization further contribute to therapy evasion [140]. As a result, novel strategies are being explored to overcome resistance and improve long-term outcomes.

One promising approach is the combination of targeted agents to block multiple pathways simultaneously. For example, ongoing clinical trials are evaluating everolimus in combination with cabozantinib, a multi-kinase inhibitor targeting VEGFR, MET, and AXL, to counteract resistance to VEGF inhibition [136]. Similarly, the dual blockade of mTOR and PI3K is under investigation as a way to prevent the feedback activation of tumor survival pathways [136]. Recent trials are evaluating alternative targeted agents for refractory PNETs. The phase III CABINET trial found that cabozantinib, a multikinase inhibitor, significantly improved PFS (13.8 vs. 4.4 months; HR 0.23, *p* < 0.001) and had a 19% response rate, outperforming prior targeted therapies [80]. However, resistance remains a challenge, and toxicity was notable (grade ≥ 3 events in ~63%). Another promising agent, surufatinib, showed improved PFS in a Chinese phase III trial, leading to its approval [136]. These findings highlight the need for newer targeted agents but underscore the persistent challenge of adaptive tumor resistance.

- **Integration of Immunotherapy**

Immunotherapy has shown limited efficacy in PNETs, as they are immunologically “cold” tumors with low TMB and poor T-cell infiltration [82]. Checkpoint inhibitors (ICIs) like pembrolizumab have response rates below 10%, and PD-L1 expression has not proven predictive [136,141]. Combination strategies are being explored, such as dual PD-1 + CTLA-4 blockade (nivolumab + ipilimumab) and anti-VEGF + ICIs (bevacizumab plus atezolizumab), which may enhance the immune response [141]. PRRT, epigenetic drugs, and cytokine therapy are also being tested to convert PNETs into “hot” tumors, potentially improving immunotherapy outcomes. High-grade pancreatic NECs, with higher mutation burdens, may be more responsive [136]. Future success will depend on integrative approaches and identifying biomarkers to guide immunotherapy use in PNETs.

- **The Role of Multi-Omics**

Advances in multi-omics have deepened our understanding of PNET biology, paving the way for personalized treatment approaches. Genomic studies confirm MEN1 mutations in 40–60% of PNETs, with DAXX/ATRX alterations linked to telomere maintenance and aggressive behavior [87]. Mutations in PTEN, TSC2, and PIK3CA highlight subsets that may benefit from mTOR inhibition, though broad genome-driven therapies remain elusive [142]. Transcriptomic analyses reveal upregulated pathways, such as PDGFRβ and CDK4/6, which are now being explored as therapeutic targets [142]. Epigenetic profiling has identified unique subtypes, including insulinomas harboring YY1 mutations, suggesting novel druggable dependencies [142]. Proteomic and metabolomic studies are uncovering biomarkers such as c-Met/AXL overexpression (which are targets of cabozantinib) and metabolic vulnerabilities like glycolysis reliance in high-grade tumors [80]. Circulating tumor DNA (ctDNA) is emerging as a promising tool for real-time genotyping and resistance monitoring [143]. While omics-driven therapy selection is not yet standard, ongoing precision oncology trials are integrating these insights, aiming to tailor treatment based on each patient’s tumor profile. As research progresses, multi-omics has the potential to transform PNET management from a generalized to a precision-based approach.

- **Disparities in Access to Care**

Access to advanced PNET treatments remains highly unequal due to their high costs and infrastructure limitations in low-resource regions. PRRT, everolimus, and Ga-68 DOTATATE PET/CT are largely restricted to high-income countries, while older therapies like interferon-α remain in use despite their poor efficacy [144]. Limited multidisciplinary expertise and a lack of specialized centers further hinder optimal care, contributing to lower resection rates and poorer outcomes, even in developed nations. Additionally, racial disparities still persist in high-income countries, with Black patients more likely to present with advanced disease and undergo fewer surgical resections, leading to worse outcomes [145]. Bridging these gaps requires expanding nuclear medicine infrastructure, reducing drug costs, and ensuring equitable participation in clinical trials to improve access and outcomes for all patients.

- **Quality of Life and Cost Considerations**

Beyond prolonging survival, maintaining patients’ quality of life (QoL) should be a critical goal in PNET management. Patients often experience chronic symptoms from the hormonal syndromes or tumor burden, with fatigue, pain, and anxiety significantly impacting daily life [146]. Fortunately, most modern therapies, including everolimus, sunitinib, and PRRT, have been shown to preserve or even improve QoL, particularly PRRT, which can alleviate symptoms in responding patients [144,147]. However, financial toxicity remains a growing concern, as targeted agents and PRRT are expensive, with limited cost-effectiveness data available. While some studies suggest that PRRT and everolimus provide reasonable value, high costs and healthcare disparities limit access, especially in low-resource settings [148]. Patients may require prolonged therapy, compounding the economic strain even in insured populations. More research is needed to evaluate the financial burden of treatment and incorporate cost-effectiveness analyses into clinical trials. Policymakers must explore value-based pricing, generic alternatives, and financial assistance programs to ensure equitable access while balancing cost sustainability in PNET care.

## 7. Conclusions

The management of pancreatic neuroendocrine tumors (PNETs) has significantly evolved beyond traditional surgery and targeted therapy, with emerging modalities offering new treatment possibilities. Peptide receptor radionuclide therapy (PRRT), immunotherapy, and novel molecular targets are reshaping the therapeutic landscape, particularly for patients with advanced or inoperable disease. Minimally invasive techniques, such as radiofrequency and microwave ablation, provide additional options for tumor control while minimizing morbidity. Despite these advancements, challenges remain in optimizing patient selection, addressing treatment resistance, and integrating novel approaches into clinical practice. A multidisciplinary approach, incorporating molecular profiling and personalized treatment strategies, is essential for improving patient outcomes. Further research is needed to refine combination therapies, expand access to innovative treatments, and enhance quality of life for individuals with PNETs. As treatment paradigms continue to evolve, a patient-centered approach will be critical in achieving long-term disease control and survival.

## Figures and Tables

**Table 1:** Classification of neuroendocrine neoplasms (NENs).

Type	Differentiation	Grade	Mitotic Rate (/10 HPF)	Ki-67 Index (%)
**NET, G1**	Well differentiated	Low	<2	<3
**NET, G2**	Well differentiated	Intermediate	2–20	3–20
**NET, G3**	Well differentiated	High	>20	>20
**NEC, small cell type**	Poorly differentiated	High	>20	>20
**NEC, large cell type**	Poorly differentiated	High	>20	>20
**MiNEN**	Well or poorly differentiated	Variable	Variable	Variable

Source: WHO Classification of Tumors of Endocrine Organs, 4th Edition, IARC 2017. Abbreviations: NET = Neuroendocrine Tumor; NEC = Neuroendocrine Carcinoma; MiNEN = Mixed Neuroendocrine–Non-Neuroendocrine Neoplasm; HPF = High-Power Field.

**Table 2:** Approach to functional PNETs.

Biochemical/Endocrine work up confirming Functional PNET		
Imaging study confirming absence of metastatic disease		
EUS-guided core needle biopsy to establish histological grade		
Insulinoma		Other functional PNET (Gastrinoma, VIPoma, Glucagonoma, ACTHoma)
Risk of metastasis and malignancy: <15%		>60%
**<2 cm**	**>2 cm or >Grade 1**	Impact of size or grade less known
Ablation vs. Surgery	Surgery	Surgery
Ablation in non-operative candidates and for post-surgical recurrence		

**Table 3:** Approach to NF-PNETs.

DOTATE Imaging to confirm absence of metastasis			
**Suspected NF PNET < 1 cm**	**Suspected NF PNET > 1–2 cm**		
No disease specific mortality at 45–65 months after diagnosis	EUS-guided Core Biopsy to confirm Histologic Grade		
	Grade 1	Grade 2	Grade 3
	3–5% LN mets	16% LN mets	>80% LN mets
Surveillance	Surveillance vs Ablation	Ablation vs Surgery	Surgery

**Table 4:** Comparison of curative surgical approaches.

Feature	Enucleation	Central Pancreatectomy	Distal Pancreatectomy	Pancreaticoduodenectomy	Total Pancreatectomy
**Typical Tumor Location**	Superficial, any location (preferably head)	Neck or proximal body	Body and tail	Head	Entire gland (diffuse or multifocal)
**Tumor Criteria**	≤2 cm, G1, not abutting main duct	≤2–3 cm, G1, non-invasive	>2 cm, G2–G3, or functional	>2 cm, G2–G3, or functional	Multifocal, recurrent, or locally advanced involving whole gland
**Functional Preservation**	Excellent	Excellent	Moderate	Low	None
**Lymph Node Dissection**	No	No	Yes	Yes	Yes
**Main Indications**	Functional tumors (e.g., insulinomas), small low-grade NF-PNETs	Low-grade tumors in neck, not amenable to enucleation	NF- or F-PNETs in distal pancreas needing oncologic clearance	Large or high-grade tumors in the head; duct involvement	MEN1 with multifocal disease, extensive tumors
**POPF Risk**	High (~30–40%)	High (20–40%)	Moderate (~20–30%)	Moderate to High (15–25%)	Comparable to PD
**Endocrine Insufficiency Risk**	Very Low	Low (~5–10%)	Moderate	High	100% (brittle diabetes)
**Exocrine Insufficiency Risk**	Very Low	Low	Moderate	High	100% (lifelong enzyme replacement)
**Preferred Surgical Approach**	Open, laparoscopic, robotic	Open, laparoscopic, robotic	Open, laparoscopic, robotic	Open, laparoscopic (limited), robotic	Open or robotic
**Limitations**	Not suitable for invasive/high-grade tumors; no nodal staging	Technically demanding, no LN dissection, higher POPF risk	Loss of tail function, possible splenectomy	High morbidity, long recovery, metabolic impact	High metabolic burden, last-resort option
